# Voxel-level biological optimisation of prostate IMRT using patient-specific tumour location and clonogen density derived from mpMRI

**DOI:** 10.1186/s13014-020-01568-6

**Published:** 2020-07-13

**Authors:** E. J. Her, A. Haworth, H. M. Reynolds, Y. Sun, A. Kennedy, V. Panettieri, M. Bangert, S. Williams, M. A. Ebert

**Affiliations:** 1 School of Physics, Mathematics and Computing, University of Western Australia, Perth, Australia; 2 Institute of Medical Physics, University of Sydney, Sydney, Australia; 3 The Sir Peter MacCallum Department of Oncology, University of Melbourne, Melbourne, Australia; 4 Department of Physical Sciences, Peter MacCallum Cancer Centre, Melbourne, Australia; 5 Department of Radiation Oncology, Sir Charles Gairdner Hospital, Perth, Australia; 6 Alfred Health Radiation Oncology, Melbourne, Australia; 7 Department of Medical Physics in Radiation Oncology, German Cancer Research Center (DKFZ), Heidelberg, Germany; 8 Department of Medical Physics in Radiation Oncology, Heidelberg Institute for Radiation Oncology, Heidelberg, Germany; 9 Division of Radiation Oncology and Cancer Imaging, Peter MacCallum Cancer Centre, Melbourne, Australia; 10 5D Clinics, Perth, Australia

**Keywords:** IMRT, Dose-painting, Tumour control probability, Multiparametric MRI, Probabilistic treatment planning

## Abstract

**Aims:**

This study aimed to develop a framework for optimising prostate intensity-modulated radiotherapy (IMRT) based on patient-specific tumour biology, derived from multiparametric MRI (mpMRI). The framework included a probabilistic treatment planning technique in the effort to yield dose distributions with an improved expected treatment outcome compared with uniform-dose planning approaches.

**Methods:**

IMRT plans were generated for five prostate cancer patients using two inverse planning methods: uniform-dose to the planning target volume and probabilistic biological optimisation for clinical target volume tumour control probability (TCP) maximisation. Patient-specific tumour location and clonogen density information were derived from mpMRI and geometric uncertainties were incorporated in the TCP calculation. Potential reduction in dose to sensitive structures was assessed by comparing dose metrics of uniform-dose plans with biologically-optimised plans of an equivalent level of expected tumour control.

**Results:**

The planning study demonstrated biological optimisation has the potential to reduce expected normal tissue toxicity without sacrificing local control by shaping the dose distribution to the spatial distribution of tumour characteristics. On average, biologically-optimised plans achieved 38.6% (*p-*value: < 0.01) and 51.2% (*p-*value: < 0.01) reduction in expected rectum and bladder equivalent uniform dose, respectively, when compared with uniform-dose planning.

**Conclusions:**

It was concluded that varying the dose distribution within the prostate to take account for each patient’s clonogen distribution was feasible. Lower doses to normal structures compared to uniform-dose plans was possible whilst providing robust plans against geometric uncertainties. Further validation in a larger cohort is warranted along with considerations for adaptive therapy and limiting urethral dose.

## Introduction

During typical radiotherapy of prostate cancer (PCa), a uniform spatial distribution of a specific dose is prescribed to the entire prostate gland, without customisation of dose prescription and distribution to the actual characteristics of an individual’s tumour. Spatial distributions of tumour characteristics may be accommodated in treatment planning through the use of non-invasive quantitative imaging. Several clinical trials that aimed to explore the feasibility of imaging-informed focal dose escalation and dose-painting have now completed (NCT01168479, NCT01208883, NCT01190527). Results of these studies are very promising [8, 37]. Nevertheless, the ways in which quantitative imaging is used in treatment planning are variable and often fail to utilise the resulting information for objective dose prescription. Many studies applied a focal boost dose to sub-volumes identified as abnormal regions on quantitative images [7, 14, 50, 60], including the FLAME-trial [37]. However, the use of sub-volumes means discretisation of tumour characteristics such as clonogen density and hypoxia, whereas typically these characteristics vary continuously throughout the gland [1, 21, 41, 42, 68]. In studies where voxel-level information was utilised, simple linear relationships between image intensities and doses were frequently assumed [3, 14, 60], or non-validated dose prescription functions were used [4, 18, 31, 38, 48, 52, 65]. Ideal biological optimisation methods require accurately defined relationships between imaging parameter, derived radiobiological parameters and validated dose-response.

To utilise the full potential of quantitative imaging in treatment planning, biofocused radiotherapy (BiRT) of PCa using the spatial distribution of image-derived tumour characteristics has been proposed [25, 26]. The proposed BiRT approach allows a simplified process of translating quantitative imaging to biologically-optimised plan using radiomics and machine learning methods. Machine learning methods, which generate voxel maps of tumour location and cell density from multiparametric magnetic resonance imaging (mpMRI), have recently been developed by our group [53, 54]. Using ‘ground truth’ histology information from a large patient database, reliable predictions from imaging can be made without the explicit understanding of underlying biological and physical processes. The voxel-level tumour information is then utilised in a tumour control probability (TCP) model that relates tumour characteristics and physical dose to the probability of tumour control. Voxel-level, patient-specific tumour information from mpMRI can be used to drive the treatment plan optimisation as model parameters to achieve a maximum TCP. Furthermore, a probabilistic treatment planning technique was adopted for biological optimisation to produce plans that are robust against geometric errors. In uniform-dose planning, a margin is used assuming that the clinical target volume (CTV) remains within the planning target volume (PTV) during irradiation in the presence of uncertainties. Therefore, giving a uniform prescription dose to a larger volume, i.e. the PTV, ensures that the prescription dose is delivered to the CTV. In contrast, the use of a margin to take account of is problematic for biological optimisation as the dose distribution is no longer uniform. Thus, probabilistic treatment planning, where the effect of geometric errors is incorporated in the expectation value of the TCP, was used to produce robust, biologically-optimised plans.

The potential of the proposed BiRT approach in producing superior treatment plans compared to conventional treatment has been demonstrated in low-dose-rate brachytherapy [9, 10, 25] using population-based clonogen distribution information and segment-level TCP model. In the current study, the BiRT approach was extended to prostate intensity-modulated radiotherapy (IMRT) planning. The purpose of the study was to test the hypothesis that a biologically-optimised prostate IMRT plan, produced using the BiRT framework, can yield reduced dose to organs at risk (OAR) compared to the isoeffective uniform-dose plan.

## Methods

### TCP model

The TCP model has been described previously for a segmented prostate in a low-dose-rate brachytherapy application [23]. Here we describe the revised model to suit voxel-level information and fractionated external beam radiotherapy. The TCP was calculated for the CTV in this study.

The radiosensitivity parameter *α* was assumed to be log-normally distributed within a population [24, 29] with a mean, $\overline{\upalpha}$ *,* and a standard deviation, σ_α_ *.* The TCP was computed using the following equation, where the distribution of *α* is normalised such that $\sum \limits_{\mathrm{k}=1}^{\mathrm{p}}\mathrm{w}\left({\upalpha}_{\mathrm{k}}\right)=1$ with *p* discrete samples:

1 $$ \mathrm{TCP}=\sum \limits_{\mathrm{k}=1}^{\mathrm{p}}\mathrm{w}\left({\upalpha}_{\mathrm{k}}\right)\mathrm{TCP}\left({\upalpha}_{\mathrm{k}}\right) $$

As a target volume consists of individual voxels, an assumption of voxel independence gives.

2 $$ \mathrm{TCP}\left({\upalpha}_{\mathrm{k}}\right)=\prod \limits_{\mathrm{i}=1}^{\mathrm{N}}\mathrm{TC}{\mathrm{P}}_{\mathrm{i}}\left({\uprho}_{\mathrm{i}},{\upalpha}_{\mathrm{k}},{\mathrm{d}}_{\mathrm{i}}\right) $$

where *TCP*_*i*_ represents the TCP of the *i* ^th^ voxel from a total of *N* voxels. *d*_*i*_ is the fractional dose to be delivered to voxel *i* and ρ_i_ represents the corresponding voxel’s clonogen density.

The voxel TCP computed with *α*_*k*_ for a fractionated treatment is described by:

3 $$ \mathrm{TC}{\mathrm{P}}_{\mathrm{i}}\left({\uprho}_{\mathrm{i}},{\upalpha}_{\mathrm{k}},{\mathrm{d}}_{\mathrm{i}}\right)=\exp \left[-{\uprho}_{\mathrm{i}}{\mathrm{V}}_{\mathrm{i}}\exp \left(-{\upalpha}_{\mathrm{k}}\mathrm{n}{\mathrm{d}}_{\mathrm{i}}-\frac{\upalpha_{\mathrm{k}}\mathrm{n}{\mathrm{d}}_{\mathrm{i}}^2}{\upalpha /\upbeta} + \ln (2)\frac{{\mathrm{T}}_{\mathrm{exp}}}{{\mathrm{T}}_{\mathrm{pot}}}\ \right)\right]\kern0.75em $$

where V_i_ represent the volume of voxel *i*. *α/β* is the alpha/beta ratio of PCa and *n* is the number of fractions, which was 39 fractions in this study with a prescription dose of 78 Gy. The presence of accelerated proliferation in PCa is still unclear with conflicting results on the effect of overall treatment time on treatment outcome [5, 19, 20, 33, 46, 63, 64]. While there may be a slight and gradual increase in repopulation with time, it is unlikely to have a significant effect as that seen in head and neck cancers. Therefore, accelerated repopulation was ignored. Clonogenic repopulation component consisted of overall treatment time, *T*_*exp*_, and potential doubling time, *T*_*pot*_. *T*_*exp*_ was approximated as 1.4*n* assuming daily fractions to be delivered during the working week. The following model parameters were derived from the work of Wang et al. [63]: $\overline{\alpha}$ =0.15 Gy^− 1^, *σ*_*α*_ =0.04 Gy^− 1^, *α*/*β* =3.1, and T_pot_ *=*42 days)*.*

### Data acquisition

A subset of five consecutive patients was selected from a cohort of PCa patients who participated in a Human Research Ethics Committee approved project (Reference number: HREC/15/PMCC/125) at the Peter MacCallum Cancer Centre, Melbourne, Australia. Informed written consent was obtained from all patients, and all underwent radical prostatectomy for their PCa management. Patient demographics are summarised in Table 1.

**Table 1:** Patient demographics

Patient number	Age (years)	PSA (ng/mL)	Gleason score of the dominant nodule	Pathological stage
1	59	16	7 (4 + 3)	pT3a
2	68	27	9 (5 + 4)	pT3b N0
3	68	10.5	7 (4 + 3)	pT3a
4	71	10	7 (3 + 4)	pT2c
5	63	11	7 (4 + 3)	pT3a

Machine learning methods have previously been developed to generate a Gaussian kernel support vector machine to predict tumour location and a general additive model to predict cell density [53, 54]. The pipeline involved the collection of in vivo mpMRI data prior to prostatectomy. MR sequences included T2-weighted, diffusion-weighted, dynamic contrast-enhanced magnetic resonance imaging (MRI) and blood-oxygen-level-dependent sequences. After prostatectomy, ex vivo MRI data were obtained from the specimens to aid co-registration of ground truth histology with mpMRI. Histology slides obtained at 5 mm intervals were annotated with tumour location and grade by an expert pathologist. Predictive models were fitted to ground truth histology and corresponding mpMRI parameters. Detailed information on the MR sequences, image registration techniques and machine learning methods used in the production of tumour biology prediction maps are contained in Reynolds et al. [49], Sun et al.[53] and Sun et al. [54].

Using the developed methods, patient-specific, voxel-level tumour location and cell density per area (number of cells/mm^2^) prediction maps were generated from mpMRI data for the five patients selected for this study.

The resolution of the tumour location and cell density per area prediction maps were 0.22 mm × 0.22 mm × 2.5 mm. To allow reduced computation time during treatment planning, the prediction maps were resampled to a voxel size of 2 mm × 2 mm × 2.5 mm.

### Clonogen distribution maps

The cell density per area prediction maps were then converted into volumetric cell density maps (number of cells/mm^3^) by raising each voxel value to the power of 3/2. Uniform cell density per area was assumed between slices. Each voxel in the tumour location prediction maps contained a continuous value between 0 and 1, representing the probability of the voxel containing tumour cells. For each patient, a threshold probability that maximised the sum of sensitivity and specificity of the receiver operating characteristics curve was selected by testing each threshold (to the nearest 0.01) incrementally. The selected threshold was then used to create a binary tumour location prediction map.

The matching binary tumour location prediction map and cell density prediction map were then multiplied together for each patient to generate a patient-specific cell distribution map. As the cell density prediction map is unable to distinguish between normal cells and tumour cells, the cell distribution maps were linearly scaled such that the median total number of cells within the prostate of the five patients was equal to 10^7^. This value represents the estimated total clonogen number of high-risk PCa patients from a study by Wang et al. [63]. Linearly scaled cell distribution maps are now referred to as clonogen distribution maps. Original and scaled cell numbers for all five patients are summarised in Table 2.

**Table 2:** The original and scaled total cell number (clonogen) for each patient

Patient	Total cell number in CTV	
	Original (normal + clonogen)	Scaled (clonogen)
1	1.81E+ 08	1.00E+ 07
2	5.41E+ 08	2.99E+ 07
3	2.22E+ 07	1.23E+ 06
4	3.83E+ 08	2.12E+ 07
5	5.93E+ 07	3.28E+ 06

### Treatment planning

Treatment planning was performed using a MATLAB based open-source program, matRad (German Cancer Research Centre, Heidelberg, Germany, version 1.4 beta) [12, 66]. matRad simulates a 6 MV linear accelerator beam using pre-calculated kernels for beam elements for user-defined beam angles. Beam element weightings are optimised by a gradient descent algorithm (version 1.4 beta) incorporating direct aperture optimisation. The original code was modified to include the biological optimisation functions and voxel-level model parameters. matRad was executed using MATLAB (version 2018b, The MathWorks Inc., Massachusetts, USA). The beamlet width was 2.5 mm and a 7-field beam geometry (0°, 40°, 80°, 110°, 250°, 280°,310°^1^) was used.

Computed tomography (CT) images were not acquired as part of the imaging protocol described in *Data acquisition*. For treatment planning, a single CT image set from an established clinical trial was selected. The selection was based on the union of the CT-defined CTV and each of the delineated CTV structures from the T2-weighted MRI of the five patients. The CTV in this study was defined as the entire prostate gland, excluding the seminal vesicles, according to ICRU Report 62 [34]. The MRI-CTV was delineated by an experienced radiation oncologist (SW). The CT image set and prediction maps were manually registered individually so that each MRI-CTV structure was completely contained within the CT-defined CTV.

#### Uniform-dose plan

The standard approach to account for uncertainties in uniform-dose planning is to apply a treatment margin to the CTV to produce the PTV. Hence for this study, we have applied a margin based on the work of van Herk et al. [27] for uniform-dose plans to account for robustness. The following formula ensures the minimum dose to the CTV is 95% of the prescription dose for 90% of the patients:

4 $$ Margin=2.5\ \varSigma +0.7\ \sigma $$

where *Σ* and *σ* represent the standard deviation of systematic and random components of geometric errors, respectively. Sources of systematic error considered in this study were target delineation and intrafraction motion [2, 32]. Treatment accuracy is also limited by fiducial marker localisation. It was assumed that systematic errors were isotropically distributed with a zero mean. A random error is any deviation that can vary in direction and magnitude for each treatment fraction. The random effect of intrafraction motion was considered in this study [32]. Sources of geometric errors and their distributions are summarised in Appendix 1. The overall geometric error distribution in each of the three principal directions with the calculated margin are summarised in Table 3.

**Table 3:** The overall treatment uncertainty distribution with the calculated margin (in mm) in three principal directions. M = mean, *Σ* = standard deviation of systematic errors, *σ* = standard deviation of random errors

Direction	M	Σ	***σ***	Margin
**AP**	−0.4	2.56	1.26	7.3
**LR**	0.2	2.47	0.67	6.6
**SI**	0.1	2.58	1.18	7.3

The dose-volume (DV) constraints and objective functions for the target volumes and OARs are summarised in Table 4. A mathematical formulation of each objective function is given in Appendix 2. The generated uniform-dose plans required PTV V74Gy (95% prescription dose) ≥ 99% and CTV V78Gy (100% prescription dose) ≥ 99%. The resulting uniform-dose plan is called Plan A.

**Table 4:** Dose-volume constraints and objective functions used in uniform-dose planning. † = Quantitative Analyses of Normal Tissue Effects in the Clinic (QUANTEC). *Radiation Therapy Oncology Group (RTOG) Consensus. Square deviation function penalises any deviation from the reference dose, D_ref_, whereas square overdose function penalises doses greater than D_ref_. Mathematical formulations of the objective functions are summarised in Appendix 2

Volume of interest	DV constraint	Objective function	Function parameter(s)
CTV	V78Gy ≥ 99%	Square deviation	D_ref_ = 78 Gy
Rectum^†^ [39]	V50Gy ≤ 50%, V60Gy ≤ 25%	DV	D_ref1_ = 50 Gy and V_ref_ = 50%, D_ref1_ = 60 Gy and V_ref_ = 25%
Bladder^†^ [61]	V65Gy ≤ 50%	DV	D_ref1_ = 65 Gy and V_ref_ = 50%
Left and right head of femurs (HOF)* [36]	V50Gy ≤ 5%	DV	D_ref1_ = 50 Gy and V_ref_ = 5%
External volume	–	Square overdose	D_ref_ = Variable to avoid hotspots

#### Biologically-optimised plan

For biological optimisation, probabilistic treatment planning technique was utilised where the effects of treatment are incorporated in the optimiser. The target objective was to maximise the expectation value of TCP. Two types of uncertainties were considered to derive robust treatment plans. The first was the uncertainty in radiosensitivity parameter, *α*, of the TCP model due to inter-patient variability. The radiosensitivity heterogeneity was applied for all patients as a log-normal distribution within a population in the calculation of TCP, as described in Eq. 1.

The second type of treatment uncertainty considered was geometric uncertainties. These were identical to those considered for uniform-dose plans with additional uncertainties introduced in MR-histology and MR-CT registration steps [13, 15, 45, 49]. Their distributions are also summarised in Appendix 1. Geometric uncertainties were integrated into the TCP objective function for biological inverse planning using the methods described in Witte et al. [67]. It was assumed that the effects of random error could be approximated by blurring the dose distribution. The effects of systematic error were approximated by the translation of the patient volume with respect to the dose matrix. The dose distribution was assumed to not change as a result of the translation and rotations were not considered. From Eqs. (1) and (2), the expectation value of TCP, <TCP>, can be written as:

5 $$ \left\langle TCP\right\rangle ={\sum}_{k=1}^p\mathrm{w}\left({\upalpha}_{\mathrm{k}}\right)\left\langle \mathrm{TCP}\left({\upalpha}_{\mathrm{k}}\right)\right\rangle $$

6 $$ \left\langle TCP\left({\alpha}_k\right)\right\rangle ={\sum}_j{G}_{Sys,j}{\prod}_i^N TC{P}_i\left({\rho}_{i,j},{\alpha}_k,{\left({G}_{rand}\otimes d\right)}_i\right) $$

*G*_*Sys*_ and *G*_*rand*_ are the Gaussian probability density functions of systematic and random errors. The summation of the overall TCP over *j* denotes integration over systematic errors. For simplicity, the value of TCP that has been integrated with probability density functions of is called an expectation or expected value of TCP. However, it should be noted that strictly speaking, this method developed by Witte et al. [67] calculates an *approximation* of the expected value under systematic and random uncertainties.

For OARs, identical dose- and DV-based constraints to uniform-dose plans (Table 4) were applied except that the expectation value of dose was substituted for absolute dose. Biologically-optimised plans required CTV <V78Gy> (volume receiving expected dose greater than or equal to 78 Gy) ≥ 99%. The resulting biologically-optimised plans are now called Plan B.

### Plan evaluation and comparison of uniform-dose and biologically-optimised dose distributions

Uniform-dose plans (Plan A) and biologically-optimised plans (Plan B) of equal <TCP> were compared by evaluating dose metrics in the five patients. To calculate <TCP>, the patient-specific clonogen distribution map was used. The corresponding sources of geometric uncertainties considered each treatment planning method was used in the <TCP> calculation.

The plans were then linearly scaled to generate a dose distribution with a < TCP> of 0.95 to allow comparison of dose to OARs between plans at the same level of expected control. A very small variation between the scaled dose distribution and the optimal solution with equal <TCP> was expected, hence we have assumed invariance of the optimal solution with dose scaling. This scaling approximates the dose escalation required to achieve equivalent tumour control, assuming any effort to reduce OAR dose at such an escalated dose would be counterproductive. Clinically relevant DV parameters and the expectation value of generalised equivalent uniform dose [43], the dose that when homogeneously given yields the same biological effect as the non-uniform dose, were calculated for the rectum and bladder. Similar to <TCP>, the expectation value of generalised equivalent uniform dose, <EUD>, was computed by approximating the effects of systematic and random error using their probability density functions:

7 $$ \left\langle EUD\right\rangle ={\sum}_j{G}_{sys,j}{\left[\frac{1}{N}{\sum}_i^N{\left({G}_{rand}\otimes D\right)}_{i,j}^a\right]}^{1/a}\kern0.5em $$

where *a* is a tissue-specific parameter (*a*_rectum_ = 6, *a*_bladder_ = 6) and *D*_*i*_ = *nd*_*i*_. For simplicity, we assumed that the shape of the rectum and bladder were invariant and that both OARs had the same intrafraction organ motion as the prostate. Mean dose to the head of femurs (HOF) and the integral energy was also determined.

Paired t-tests were performed for the comparison between dose and DV parameters of isoeffective Plans A and B, with the R statistical language (R Foundation for Statistical Computing, Austria, Version 3.2.3). A test statistic (*p*) less than 0.05 was considered significant.

## Results

**Fig. 1:**
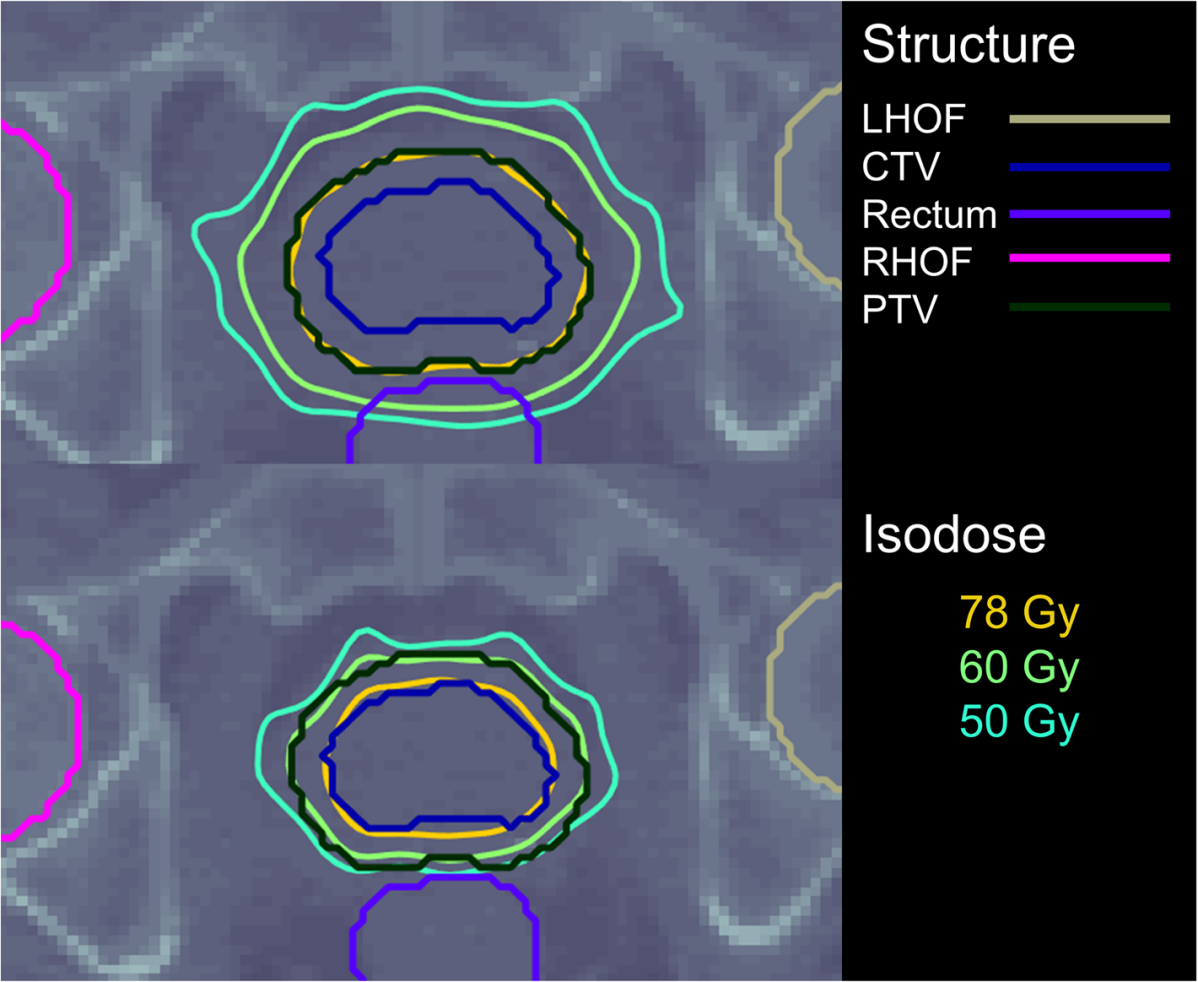
Isodose distributions for Patient 1. Above: Uniform-dose plan, Plan A. Below: Biologically-optimised plan, Plan B. Isodose distributions for other patients are available in the supplementary document

**Fig. 2:**
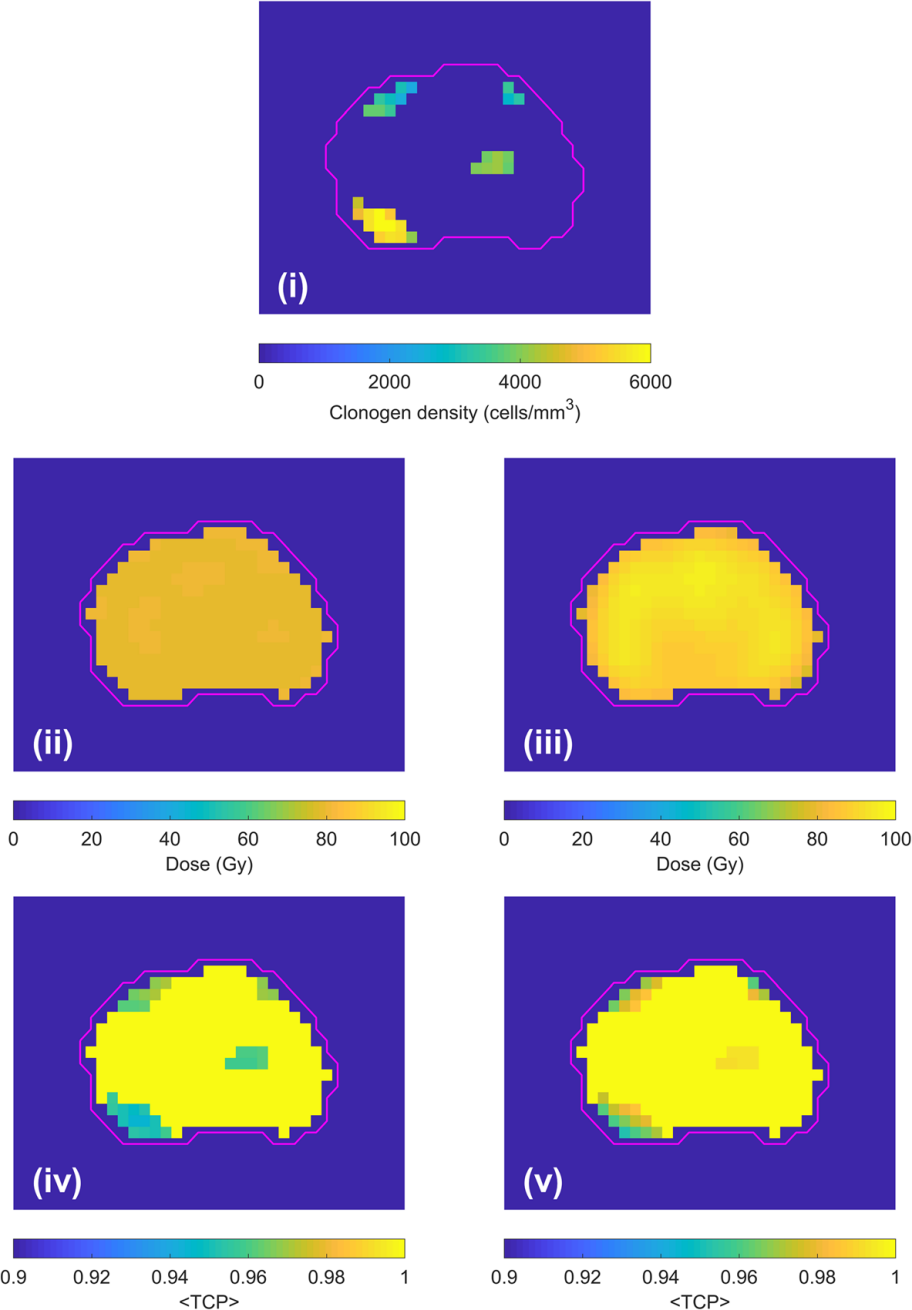
Treatment plan data for Patient 1. Axial slice corresponds to Fig. 1. Magenta contour represents the CTV. (i) clonogen distribution map. (ii-iii) CTV dose distribution of the Plans A and B. (iv-v) Corresponding <TCP> distribution of the Plans A and B. Results for all patients are available in the supplementary document

The optimised dose distributions on identical axial slices for Plans A and B are presented for Patient 1 in Fig. 1. The corresponding clonogen distribution maps, CTV dose and < TCP> distributions are displayed in Fig. 2. Resulting dose metrics of each plan are summarised in Table 5.

**Table 5:** Dose parameters for isoeffective Plans A and B. Dose distributions of the plans were scaled to achieve equivalent tumour control (〈TCP〉 = 0.95)

Dose parameters		Patient 1		Patient 2		Patient 3		Patient 4		Patient 5		Mean absolute change (mean % change)	***p***
		Plan A	Plan B	Plan A	Plan B	Plan A	Plan B	Plan A	Plan B	Plan A	Plan B		
〈**TCP**〉		0.70	0.75	0.60	0.73	0.84	0.88	0.61	0.78	0.75	0.90	+0.11 (+ 16.3%)	**0.02**
〈**TCP**〉**=0.95 scaled**	**CTV** **D**_**mean**_ **(Gy)**	100.0	108.3	104.2	112.0	91.9	103.8	102.9	115.0	95.8	101.6	+ 9.2 (+ 9.3%)	**< 0.01**
	**PTV** **D**_**mean**_ **(Gy)**	100.0	88.9	104.2	93.4	91.9	87.1	103.0	91.7	95.8	86.2	−9.5 (−9.5%)	**<0.01**
	**Rectum** **V60 (%)**	4.4	0.2	3.2	0.1	1.1	0.0	1.0	0.0	1.6	0.2	−2.2 (−96.0%)	**0.03**
	**Rectum** **V50 (%)**	6.7	0.7	4.8	0.6	2.4	0.1	1.8	0.1	2.6	0.4	−3.3 (−90.4%)	**0.01**
	**Bladder** **V65 (%)**	3.1	0.0	1.9	0.0	3.1	0.0	0.8	0.0	2.6	0.0	−2.3 (−100%)	**<0.01**
	**Rectum** 〈**EUD**〉 **(Gy)**	47.7	29.1	45.5	27.1	35.1	22.2	36.4	21.5	39.9	25.6	−15.8% (−38.6%)	**<0.01**
	**Bladder** 〈**EUD**〉 **(Gy)**	46.9	21.9	43.4	22.8	44.0	23.1	36.2	14.1	43.7	23.3	−21.8 (−51.2%)	**<0.01**
	**Left HOF** **D**_**mean**_ **(Gy)**	22.7	18.0	22.4	15.8	21.8	14.0	22.3	13.1	22.4	16.0	−6.9 (−31.2%)	**<0.01**
	**Right HOF D**_**mean**_ **(Gy)**	20.7	14.7	21.1	12.1	20.0	13.8	19.9	11.3	21.2	13.5	−7.5 (−36.4%)	**<0.01**
	**Integral energy (× 10** ^**6**^ **J/cm** ^**3**^ **)**	10.00	6.05	9.45	5.56	9.30	5.95	9.14	4.99	9.57	5.78	−3.8 (−40.3%)	**<0.01**

**Fig. 3:**
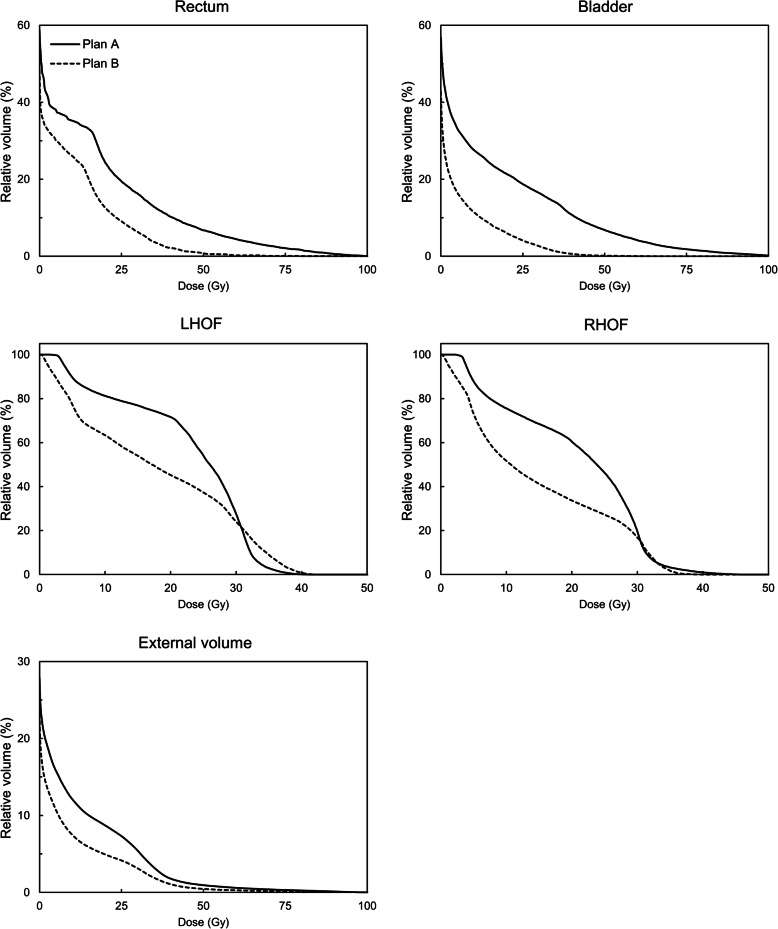
OAR DVH for Patient 1, isoeffective Plans A and B. Dose distributions of the plans were scaled to achieve equivalent tumour control (<TCP > =0.95). Results for all patients are available in the supplementary document

**Fig. 4:**
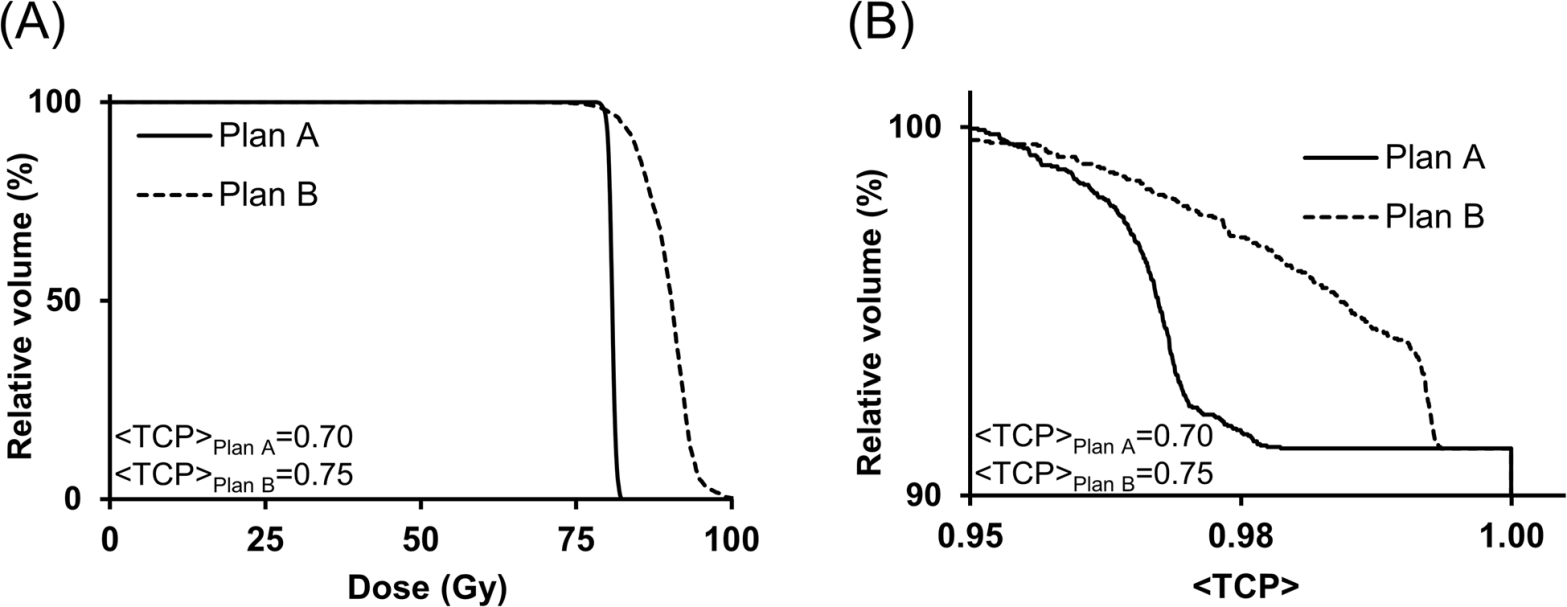
(A) CTV DVH and (B) < TCP>-volume histogram for Plans A and B of Patient 1

 With biological optimisation, higher dose to the CTV (*p*-value: < 0.01) was achieved with significantly lower <EUD> to the rectum (*p*-value: < 0.01) and bladder (*p*-value: < 0.01) for the isoeffective uniform-dose plans (Table 5 and Fig. 3) and well within the dose constraints. Plan B demonstrated substantial improvement in rectal and bladder NTCP. Due to a high variance in NTCP values for Plan A, attributed to high sensitivity of the model in the given <EUD> range, statistical significance could not be demonstrated (available in the supplementary document). A statistically significant reduction in mean doses to the HOFs was achievable (*p*-value:< 0.01). It is also evident from that biological optimisation was successful in modulating the beam intensities within the CTV, following the required dose dictated by the varying clonogen densities whilst accounting for treatment uncertainties. As a result, on average, biologically-optimised Plan B achieved 16.3% greater <TCP> than Plan A (*p*-value: 0.02). The biofocused approach improved the <TCP> by directly incorporating <TCP> evaluation in the treatment plan optimisation algorithm. Plan B could achieve a significantly higher <TCP> in all five patients, by increasing the value of individual voxel <TCP> and < TCP> homogeneity (Fig. 2 and Fig. 4).

## Discussion

A framework for optimising prostate IMRT with mpMRI-derived patient-specific tumour characteristics with probabilistic treatment planning technique has been presented. Uniform-dose and biologically-optimised IMRT plans for five patients were generated and compared. Whilst our sample size was small, this study illustrated the potential advantage of biological optimisation in yielding an improved expected probability of tumour control while achieving better sparing of OARs. Future studies will incorporate a larger sample size with a range of tumour volumes and tumour position within the prostate to assess the variation in benefit of the proposed approach. It is anticipated that these studies will identify key clinical features that will predict the benefit of the proposed BiRT approach.

Under the assumption of voxel independence, a maximum TCP is attained when the TCP for the individual voxels across the PTV are high and spatially homogeneous [17]. In the presence of tumour heterogeneity due to varying clonogen density or other relevant parameters, a uniform-dose distribution will have a highly non-uniform TCP distribution (Fig. 2 (iv)). By directly incorporating TCP in treatment planning optimisation, the ability of IMRT to modulate the beam intensity within the CTV is realised (Fig. 2 (v)). An increase in dose inhomogeneity was observed in the biologically-optimised plans (Plan B). On average, Plan B demonstrated a 11-percentage point improvement (*p*-value:0.02) in <TCP> compared to Plan A which was based on physical dose optimisation. A relatively lower <TCP> around the periphery of the CTV is most likely due to the physical restriction of the IMRT dose gradient. The ability to modulate beam intensity with clonogen distribution allowed a lower dose to the rectum and bladder in pursuit of the overall tumour control objective. When Plans A and B with an equal <TCP> of 0.95 were compared, Plan B demonstrated 38.6% (*p*-value:< 0.01) and 51.2% (*p*-value:< 0.01) reduction in rectum and bladder <EUD>, on average. With full-biological optimisation where probabilistic objectives such as <NTCP> and < EUD> minimisation are applied to rectum and bladder, a treatment plan with minimal predicted tissue toxicity could be achieved.

The standard of care of PCa with IMRT is moving away from uniform-dose planning with the progressive evolvement of quantitative imaging. Dose escalation in regions informed by imaging is becoming more common in the clinical setting. However, the majority of the dose-escalation is applied to sub-volumes that are manually contoured based on image intensity, leading to large variations in sub-volumes [51]. To overcome this limitation, investigators used a dose-painting-by-numbers technique where the dose prescription is applied at the voxel level [6, 8, 14, 16, 22, 44, 52, 57, 69]. Rather than using a dose-prescription approach, we have applied a voxel-level TCP objective function to incorporate the expected treatment outcome. Furthermore. unlike previous studies that assumed clonogens are evenly distributed within the tumour or that all patients have an identical number of clonogens and/or radiosensitivity [16, 57, 58, 67], the proposed BiRT approach accounts for tumour heterogeneity derived from mpMRI.

The proposed biofocused approach provides robust solutions by incorporating treatment-related uncertainties in the optimisation process. In this study, the expectation value of TCP was optimised using the method developed by Witte et al. [67]. Probabilistic treatment planning techniques remove the need for expansion of CTV into PTV and have demonstrated improved robustness to margin-based treatment planning methods [11, 40, 59, 62]. However, there are limitations to overcome before clinical implementation of margin-less probabilistic treatment planning becomes a reality. As with all probabilistic treatment planning, the accuracy of geometric uncertainty distributions is critical to the treatment outcome, even more so than those associated with radiobiological models [67]. Since a sharp dose gradient is dictated by the probabilistic approach [67] as well as an inhomogeneous clonogen distribution, an underestimation of the geometric errors, especially the systematic component, may result in treatment failure. While the current work adopted a complete margin-less approach, a small margin may be necessary to accommodate uncertainties that were not considered. There may be clonogens outside of the CTV due to nodal involvement or other geometric uncertainties that have not been accounted for. Probabilistic treatment planning is also more computationally intensive than dose-based methods due to complex objective functions. To speed up the process, a compromise had to be made in the computation matrix resolution which was initially planned for 1 mm × 1 mm × 2.5 mm but reduced to 2 mm × 2 mm × 2.5 mm. Similarly, Witte et al. used a 4 mm × 4 mm × 4 mm dose grid to enable faster computation process. As the cost of computing power is reduced, it is expected that probabilistic biological optimisation will be widely adopted while preserving high-resolution data provided by quantitative and multiparametric imaging.

The utilised TCP model has limitations in describing the complex nature of dose-response. Our model assumes the distribution of clonogen density remains constant during the treatment when it is expected to change as treatment progresses in reality. Methods to monitor such response are currently unavailable, however, studies are underway (for example Clinical trial ANZCTR UTN U1111–1221-9589) to investigate the potential to model such response using mpMRI. With this information, the TCP model could be extended to account for changes in tumour biology characteristics in response to treatment. Our TCP model similarly excludes the effect of hypoxia, in model parameters and the inherent assumption that all clonogens must be eradicated to achieve tumour control in the absence of reliable information available for modelling. Thus, the calculated TCP values can only be considered in the relative rather than absolute sense. Therefore, whilst a better outcome is predicted in biologically-optimised plans when compared with the uniform-dose approach, the absolute probability of tumour control cannot be quantified in this study. The uncertainty in radiosensitivity parameter, *α*, arising from inter-patient variability was accounted for applying a log-normal population distribution. In practice, inter- and intra-tumour heterogeneity in radiosensitivity for the individual patient will be presented in the form of a 3D biological map containing a distribution of Gleason score and hypoxia throughout the tumour generated from mpMRI using biomarkers formulated by Sun et al. [55, 56].

Uncertainties in the models to predict tumour location and cell density have been previously quantified [53, 54]. Tumour location threshold uncertainty was found to have an insignificant effect on the total clonogen number. Cell density has a linear relationship with TCP between the range defined by the 95% confidence intervals of the parameter. Hence, the predicted clonogen density values were considered as the expected values and no further data manipulation was performed to incorporate the uncertainties in clonogen density prediction map.

Furthermore, this study did not consider the urethra in treatment planning optimisation. Despite advances in dose delivery techniques, urethral strictures remain one of the most serious side-effects of prostate radiotherapy [28, 30, 35, 47], with reported incidences up to 20% [28]. As the urethra could not be easily delineated in the MR datasets, it was not possible to spare the urethra in the optimisation process. Future studies will model the urethra position using the ground truth histology from the BiRT cohort, and hence urethra sparing techniques may be possible. A randomized clinical trial that aimed to spare the MRI-defined urethra in prostate IMRT failed to improve urinary quality of life (Vainshtein et al. 2012) while delivering a uniform distribution of dose to the prostate. The proposed BiRT approach has the potential to spare the prostatic urethra while maintaining a high tumour control where the prostate is no longer subjected to a uniform-dose. However, such an approach requires sophisticated image-guided treatment delivery that can verify the position of the urethra prior to (and potentially during) treatment. Further work in this area is required for the establishment of an appropriate urethral margin.

## Conclusion

This planning study has compared uniform-dose plans with biologically-optimised IMRT plans for five PCa patients. The proposed biofocused approach utilises patient-specific tumour location and clonogen density information derived from mpMRI using a probabilistic treatment optimisation approach. Results have demonstrated that for an equivalent level of expected tumour control, a reduction in rectal and bladder dose can be achieved with the proposed BiRT methods in comparison with uniform-dose treatment planning methods.

### Supplementary information

**Additional file 1.**

## Data Availability

The datasets generated during and/or analysed during the current study are available from the corresponding author on reasonable request.

## Appendix 1: Geometric errors (in mm)

Target delineation [2].

Σ = 2 mm in all directions, M = 0 mm.

Fiducial marker localisation [32].

Σ =1 mm in all directions, M = 0 mm.

Intrafraction motion [32].

**Table:**

	M	Σ	***σ***
AP	−0.4	1.25	1.26
LR	0.2	1.05	0.67
SI	0.1	1.28	1.18

MR to histology registration [49].

Σ =3.3 mm in all directions, M = 0 mm.

MR to CT registration [13, 15, 45].

Σ =2 mm in all directions, M = 0 mm

## Appendix 2: Objective functions

*H*(*x*) is a Heaviside step function:

$$ H(x)\left\{\begin{array}{c}0,x<0\\ {}1,x\ge 0\end{array}\right. $$

*W* and *N* are the assigned penalty (weighting) and the voxel number of the volume of interest.

*Square overdose*

$$ F=\frac{W}{N}\sum \limits_{i=1}^NH\left({D}_i-{D}_{ref}\right){\left({D}_i-{D}_{ref}\right)}^2 $$

*Square underdose*

$$ F=\frac{W}{N}\sum \limits_{i=1}^NH\left({D}_{ref}-{D}_i\right){\left({D}_{ref}-{D}_i\right)}^2 $$

*Square deviation*

$$ F=\frac{W}{N}\sum \limits_{i=1}^N{\left({D}_i-{D}_{ref}\right)}^2 $$

*Dose-volume.*

*D*_*ref*1_, *V*_*ref*_ = desired dose-volume constraint.

*D*_*ref*2_ = current evaluation of the dose corresponding to *V*_*ref*_

$$ F=\frac{W}{N}\sum \limits_{i=1}^NH\left({D}_{ref2}-{D}_i\right)\ H\left({D}_i-{D}_{ref1}\right){\left({D}_i-{D}_{ref1}\right)}^2 $$

*Maximise < TCP>*

$$ F=\frac{W}{N}\times -\ln \left(\left\langle TCP\right\rangle \right) $$

## Abbreviations

BiRT: Biofocused radiotherapy
CT: Computed tomography
CTV: Clinical target volume
DV: Dose-volume
EUD: Equivalent uniform dose
H&E: Haematoxylin and Eosin
IMRT: Intensity-modulated radiotherapy
mpMRI: Multiparametric MRI
MRI: Magnetic resonance imaging
OAR: Organ at risk
PTV: Planning target volume
TCP: Tumour control probability

## Footnotes

1 International Electrochemical Commission definitions
