# Supplementary material for: Voxel-level biological optimisation of prostate IMRT using patient-specific tumour location and clonogen density derived from mpMRI
Source: Radiat Oncol. 2020 Jul 13;15:172. doi: 10.1186/s13014-020-01568-6 (PMC7805066; doi:10.1186/s13014-020-01568-6)
Supplement: Supplementary file 1 — Additional file 1. [file 13014_2020_1568_MOESM1_ESM.docx]

**Supplementary to “Voxel-level biological optimisation of prostate IMRT using patient-specific tumour location and clonogen density derived from mpMRI”**


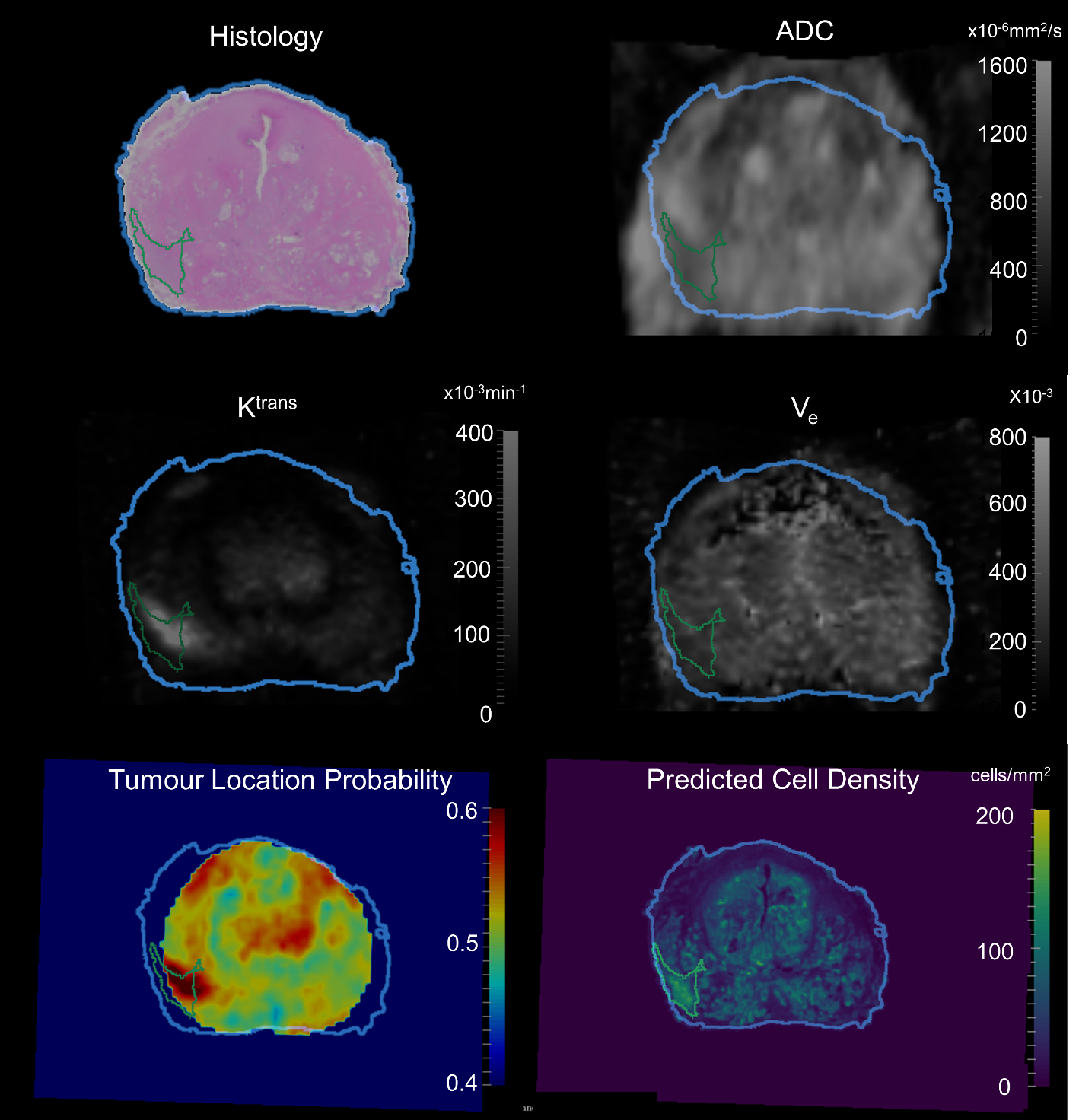


Figure 1 Haematoxylin and Eosin stained histology slice, selected image parameter and predicted tumour biology characteristics maps for Patient 1. Light blue contour represents the prostate and green contour represents tumour location drawn by a pathologist. ADC=Apparent Diffusion Coefficient from diffusion-weighted images, K^trans^=volume transfer constant from dynamic contrast-enhanced images and V_e_=interstitial volume from dynamic contrast-enhanced images.


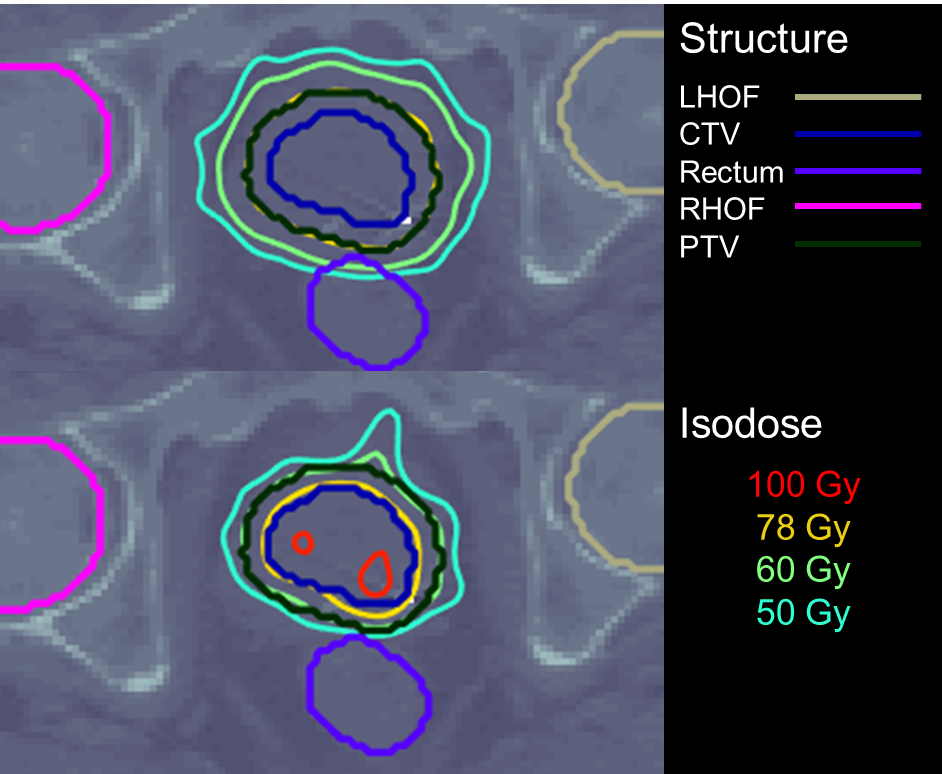


Figure 2 Isodose distributions for Patient 2. Above: Uniform-dose plan, Plan A. Below: Biologically-optimised plan, Plan B.


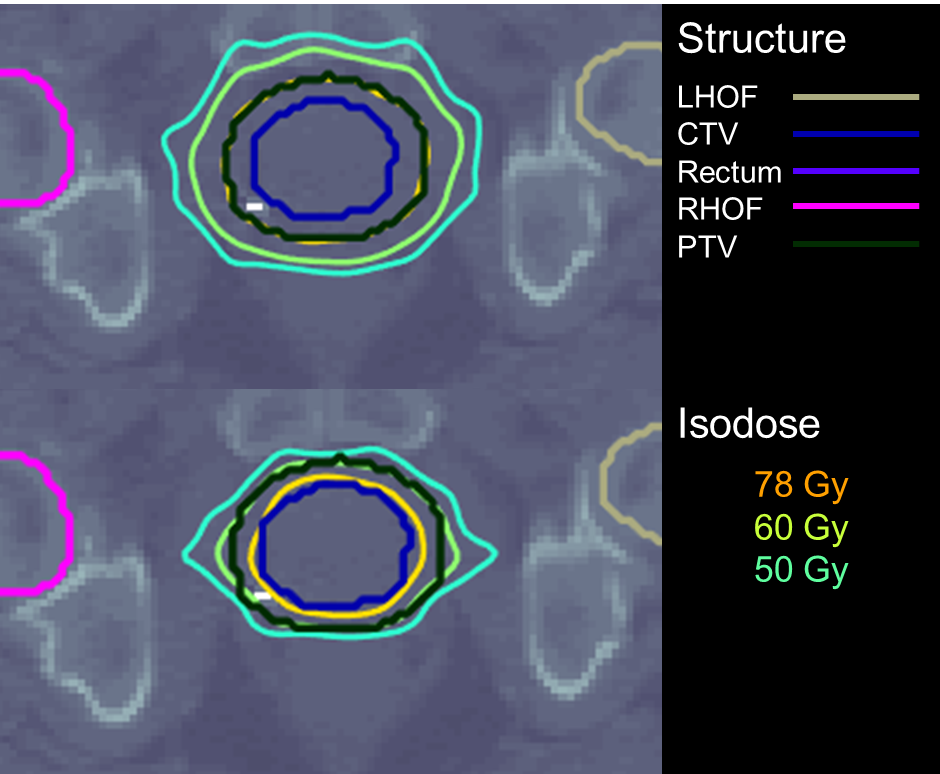


Figure 3 Isodose distributions for Patient 3. Above: Uniform-dose plan, Plan A. Below: Biologically-optimised plan, Plan B.

*
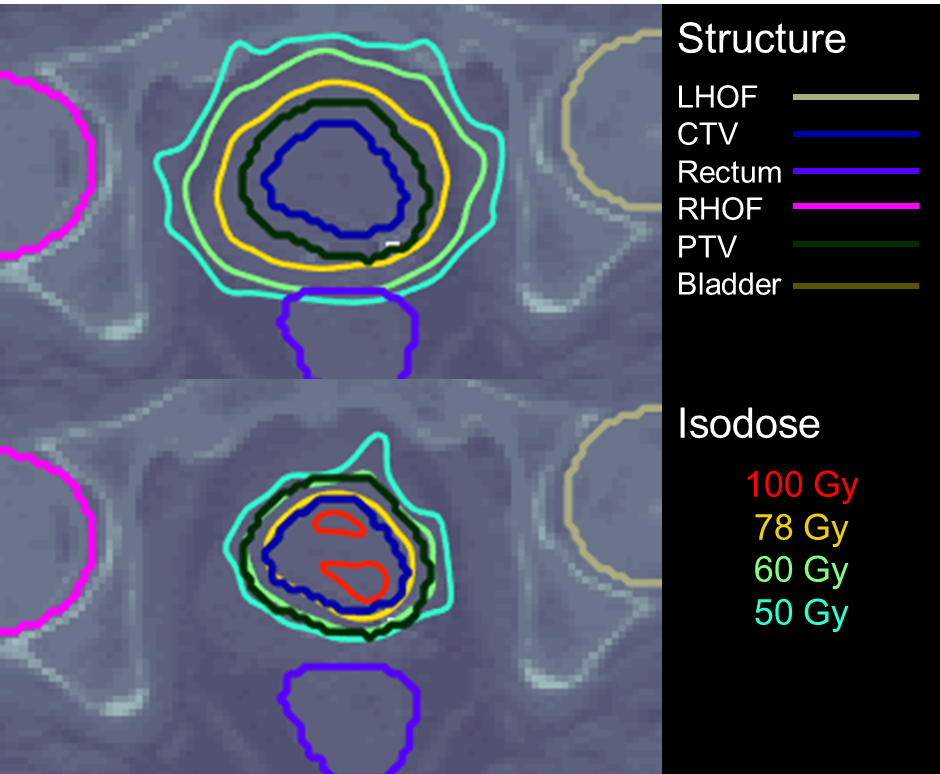
*

Figure 4 Isodose distributions plans for Patient 4. Above: Uniform-dose plan, Plan A. Below: Biologically-optimised plan, Plan B.


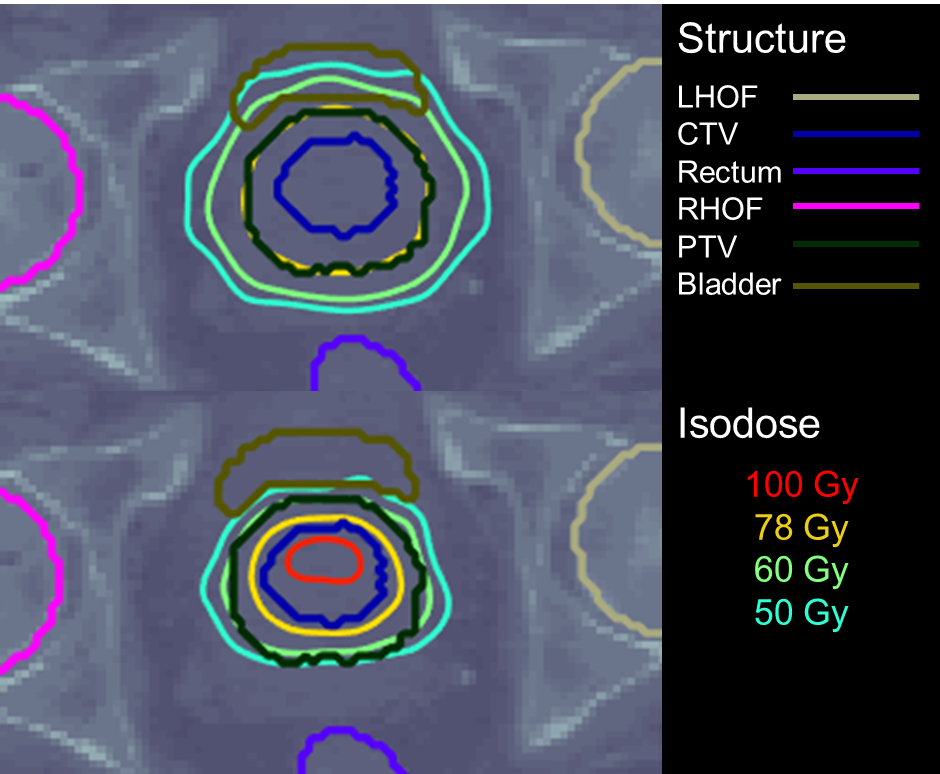


Figure 5 Isodose distributions for Patient 5. Above: Uniform-dose plan, Plan A. Below: Biologically-optimised plan, Plan B.


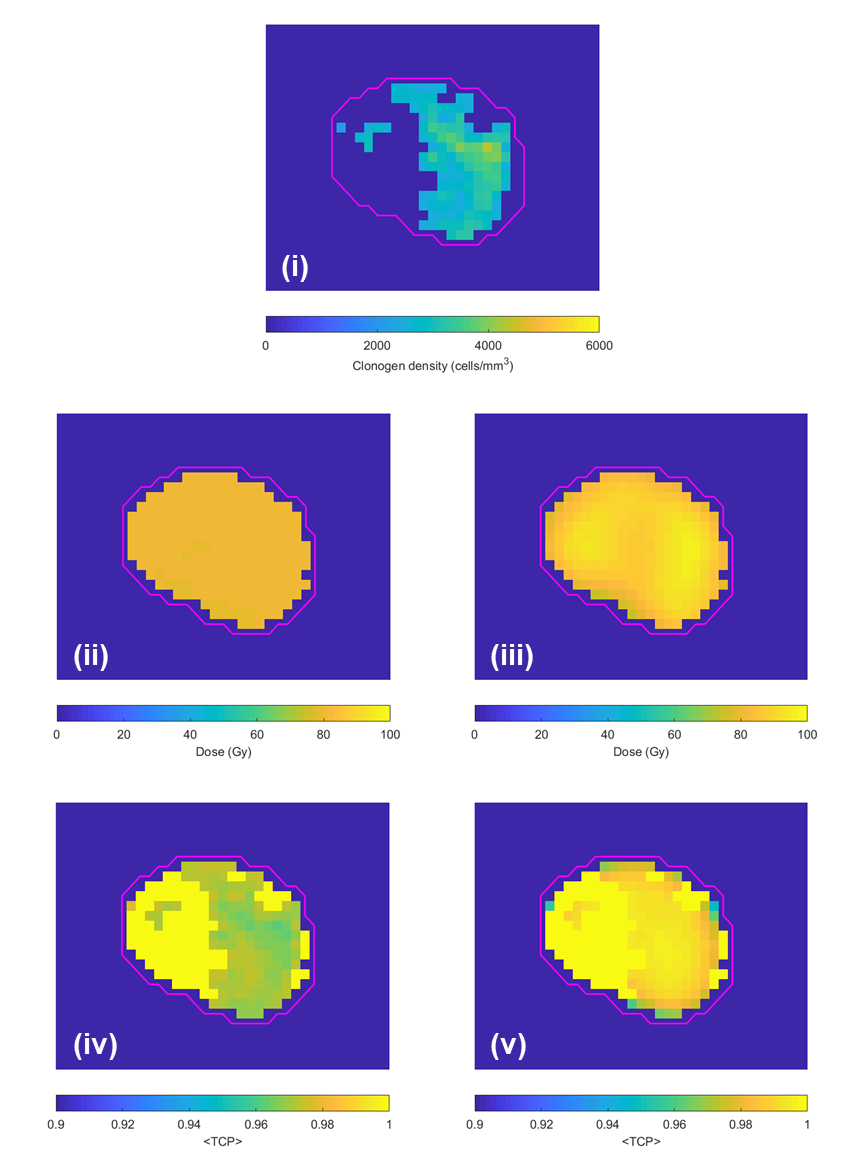


Figure 6 Treatment plan data for Patient 2. Axial slice corresponds to Figure 2. Magenta contour represents the CTV. (i) clonogen distribution map. (ii-iii) CTV dose distribution of the Plans A and B. (iv-v) Corresponding <TCP> distribution of the Plans A and B.


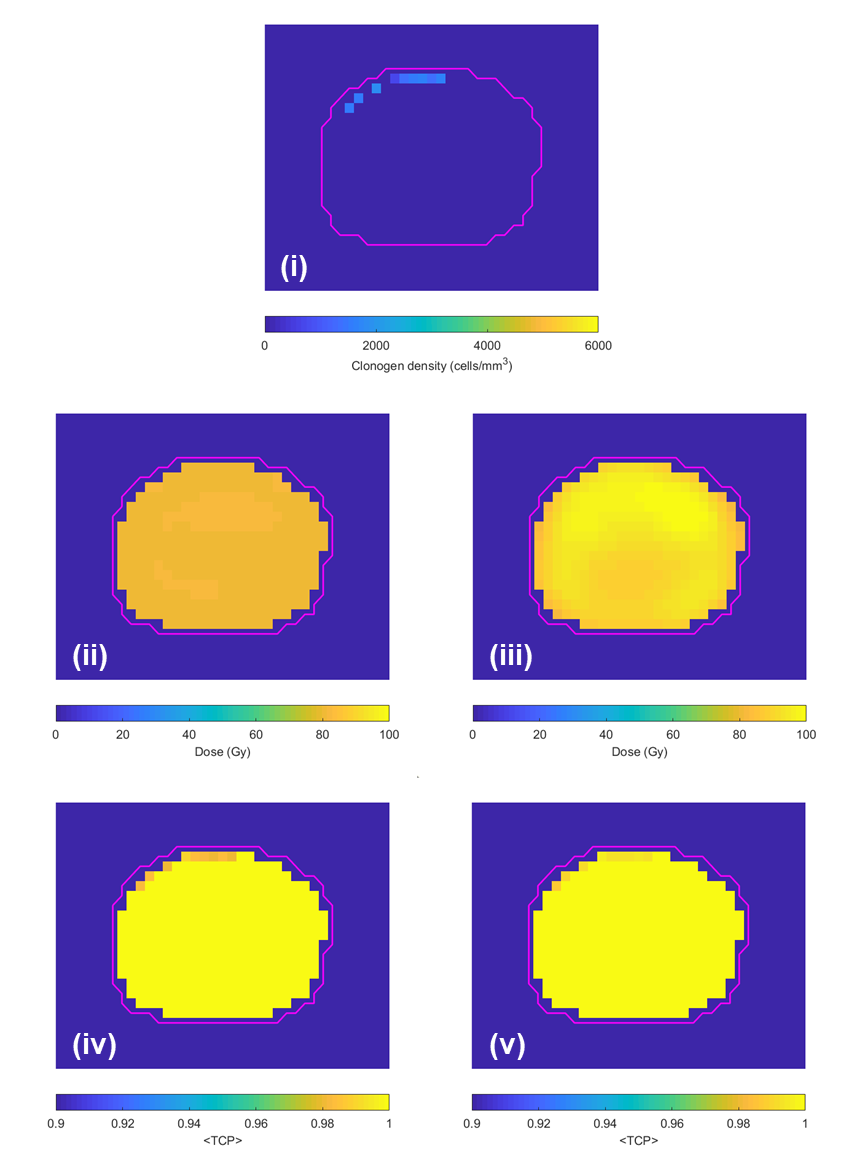


Figure 7 Treatment plan data for Patient 3. Axial slice corresponds to Figure 3. Magenta contour represents the CTV. (i) clonogen distribution map. (ii-iii) CTV dose distribution of the Plans A and B. (iv-v) Corresponding <TCP> distribution of the Plans A and B.


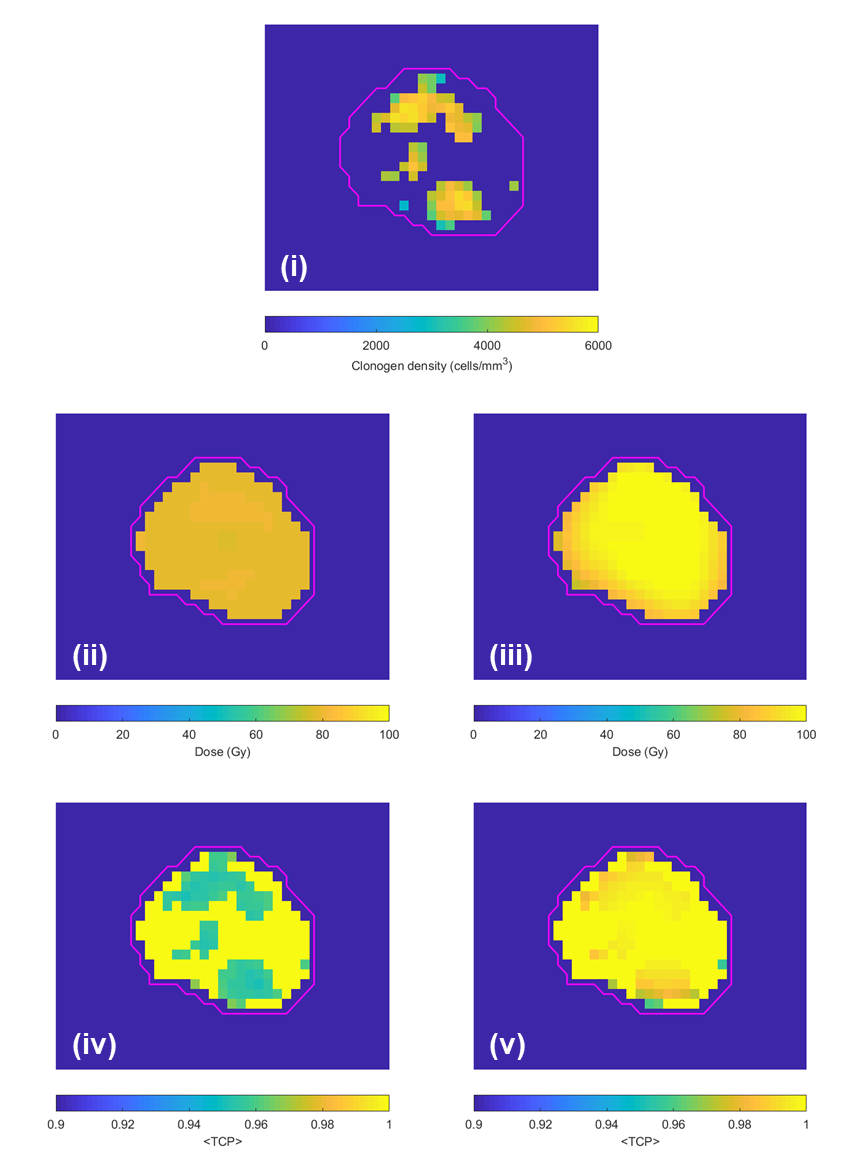


Figure 8 Treatment plan data for Patient 4. Axial slice corresponds to Figure 4. Magenta contour represents the CTV. (i) clonogen distribution map. (ii-iii) CTV dose distribution of the Plans A and B. (iv-v) Corresponding <TCP> distribution of the Plans A and B.


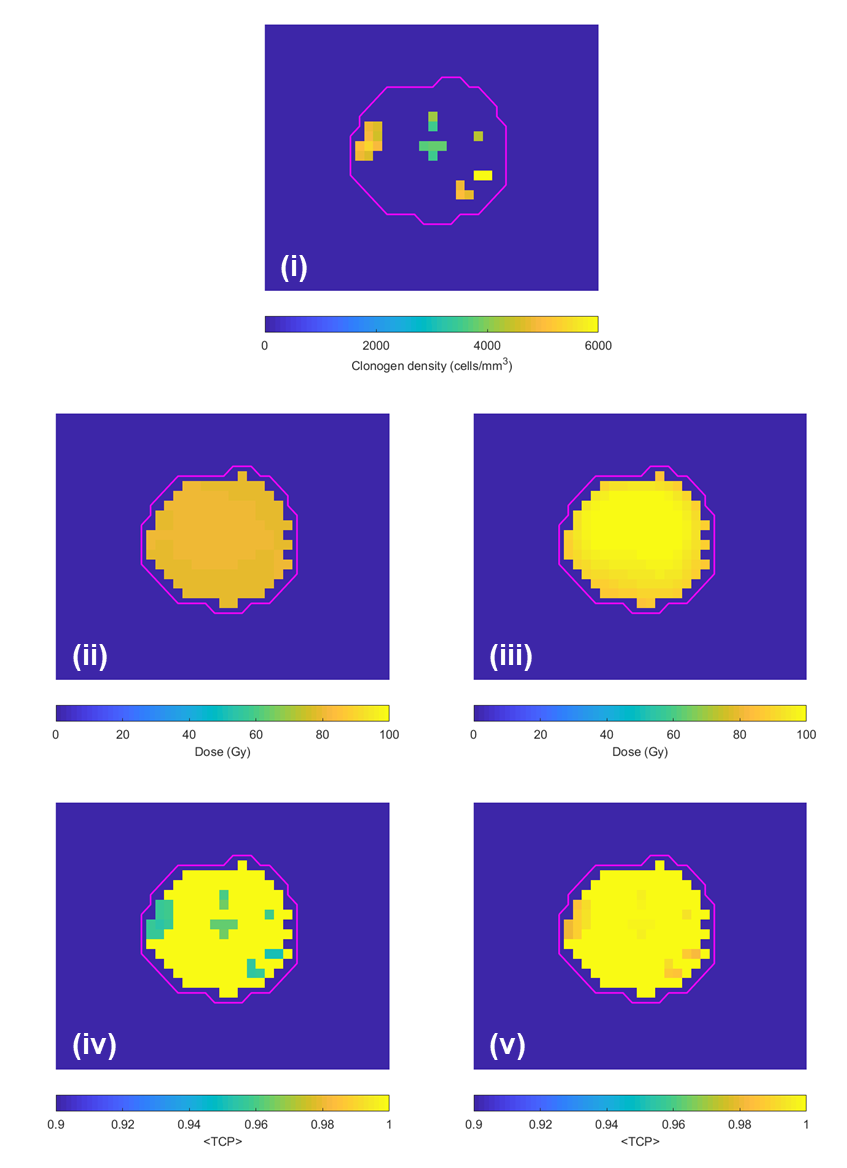


Figure 9 Treatment plan data for Patient 5. Axial slice corresponds to Figure 5. Magenta contour represents the CTV. (i) clonogen distribution map. (ii-iii) CTV dose distribution of the Plans A and B. (iv-v) Corresponding <TCP> distribution of the Plans A and B.


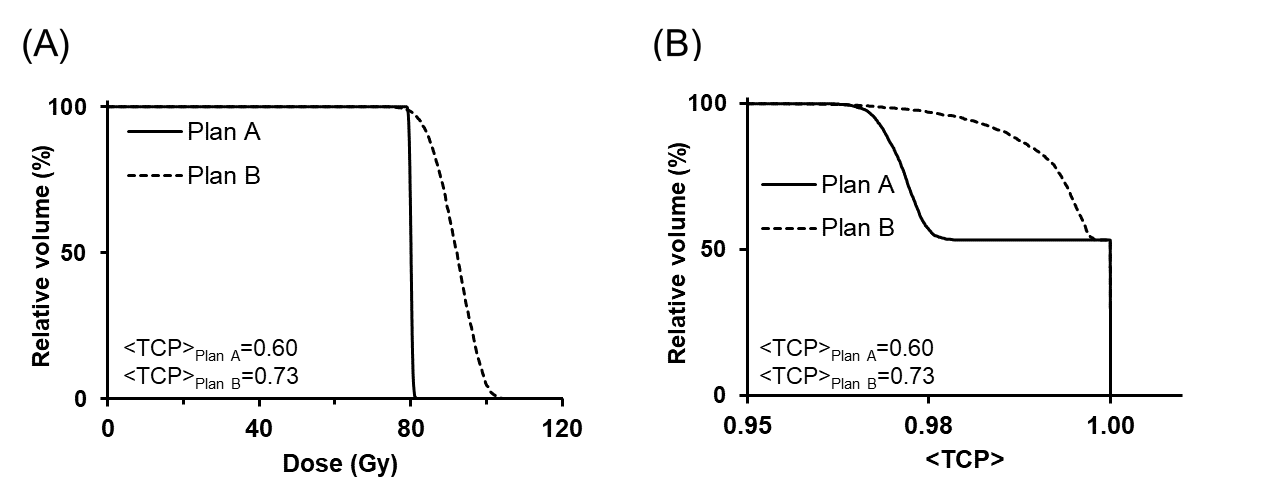


Figure 10 (A) CTV DVH and (B) <TCP>-volume histogram for Plans A and B of Patient 2.


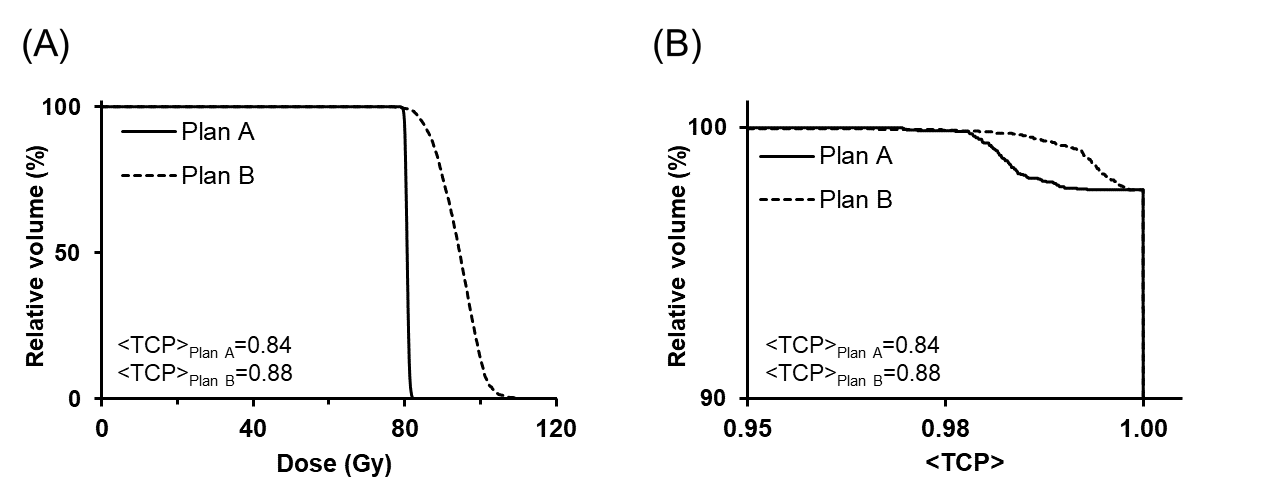


Figure 11 (A) CTV DVH and (B) <TCP>-volume histogram for Plans A and B of Patient 3.


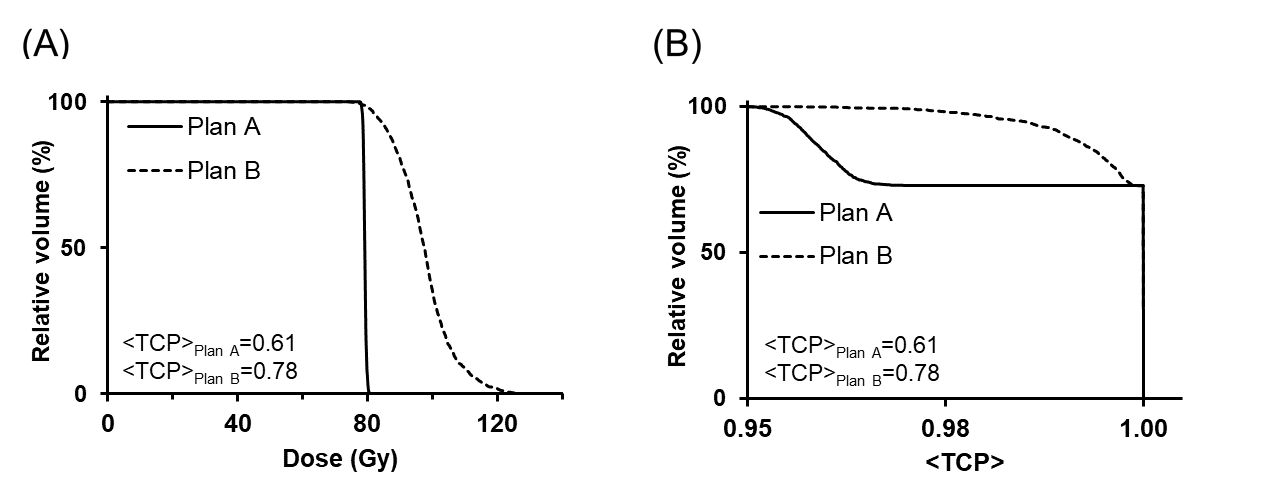


Figure 12 (A) CTV DVH and (B) <TCP>-volume histogram for Plans A and B of Patient 4.


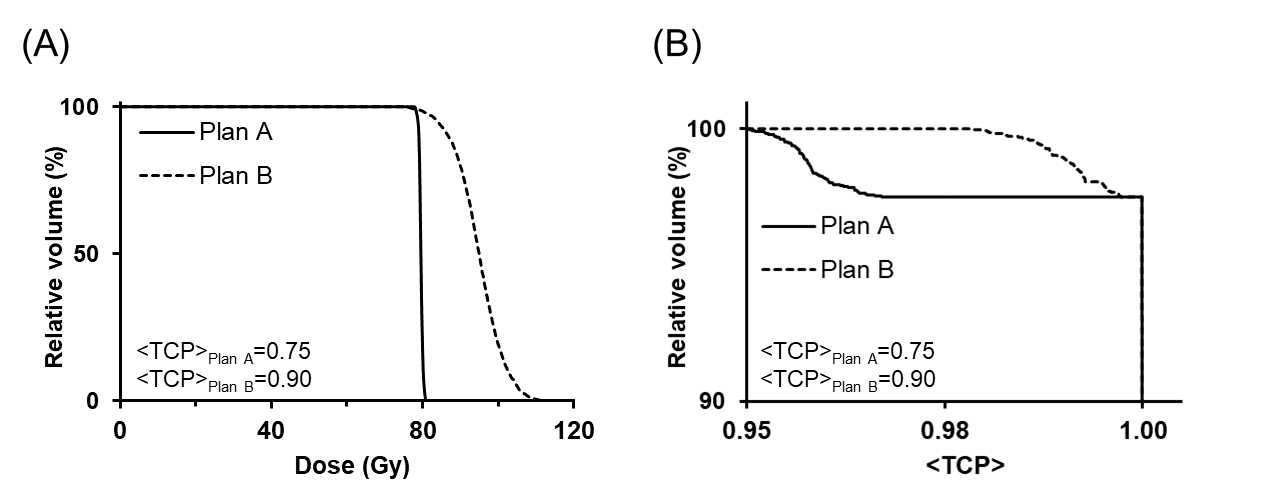


Figure 13 (A) CTV DVH and (B) <TCP>-volume histogram for Plans A and B of Patient 5.


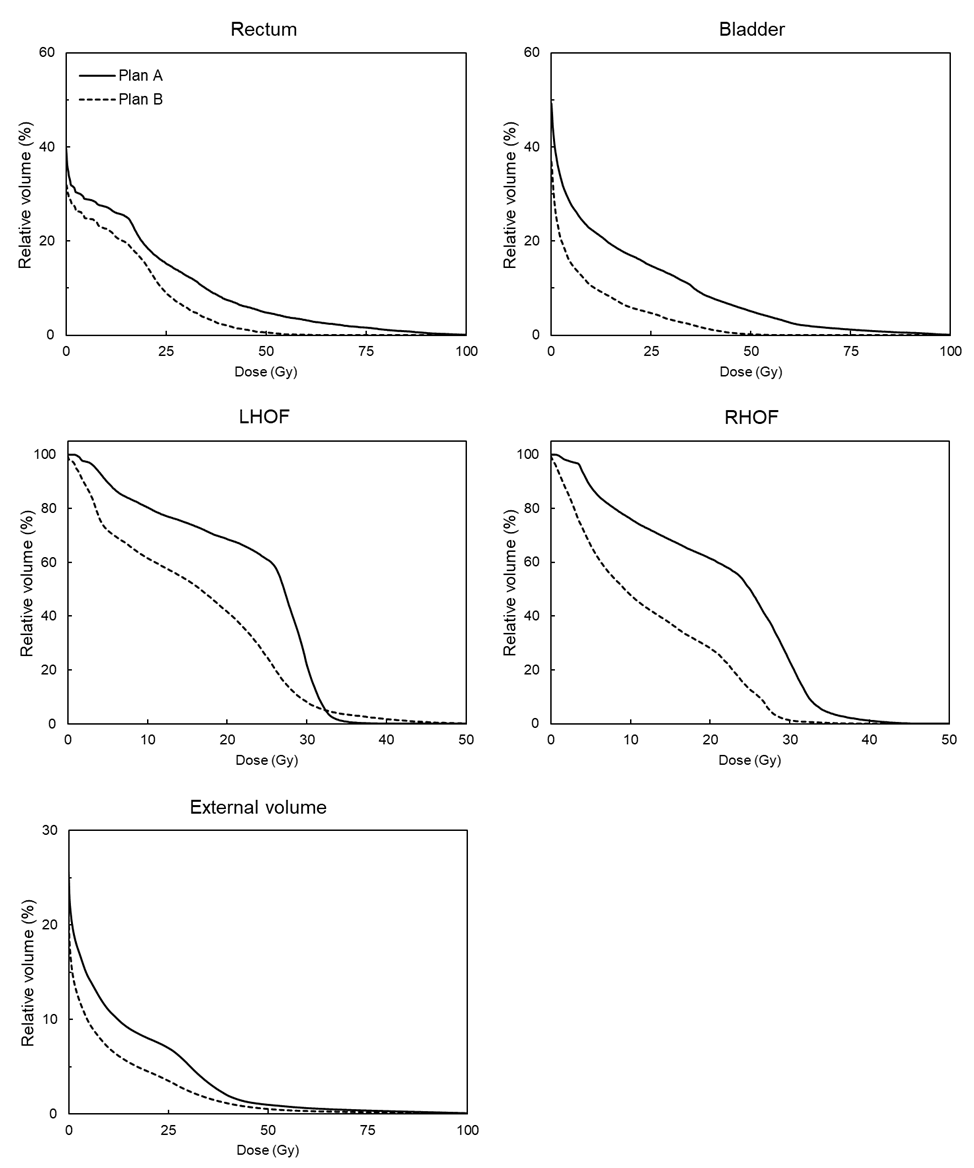


Figure 14 OAR DVH for Patient 2, isoeffective Plans A and B with a <TCP>=0.95.


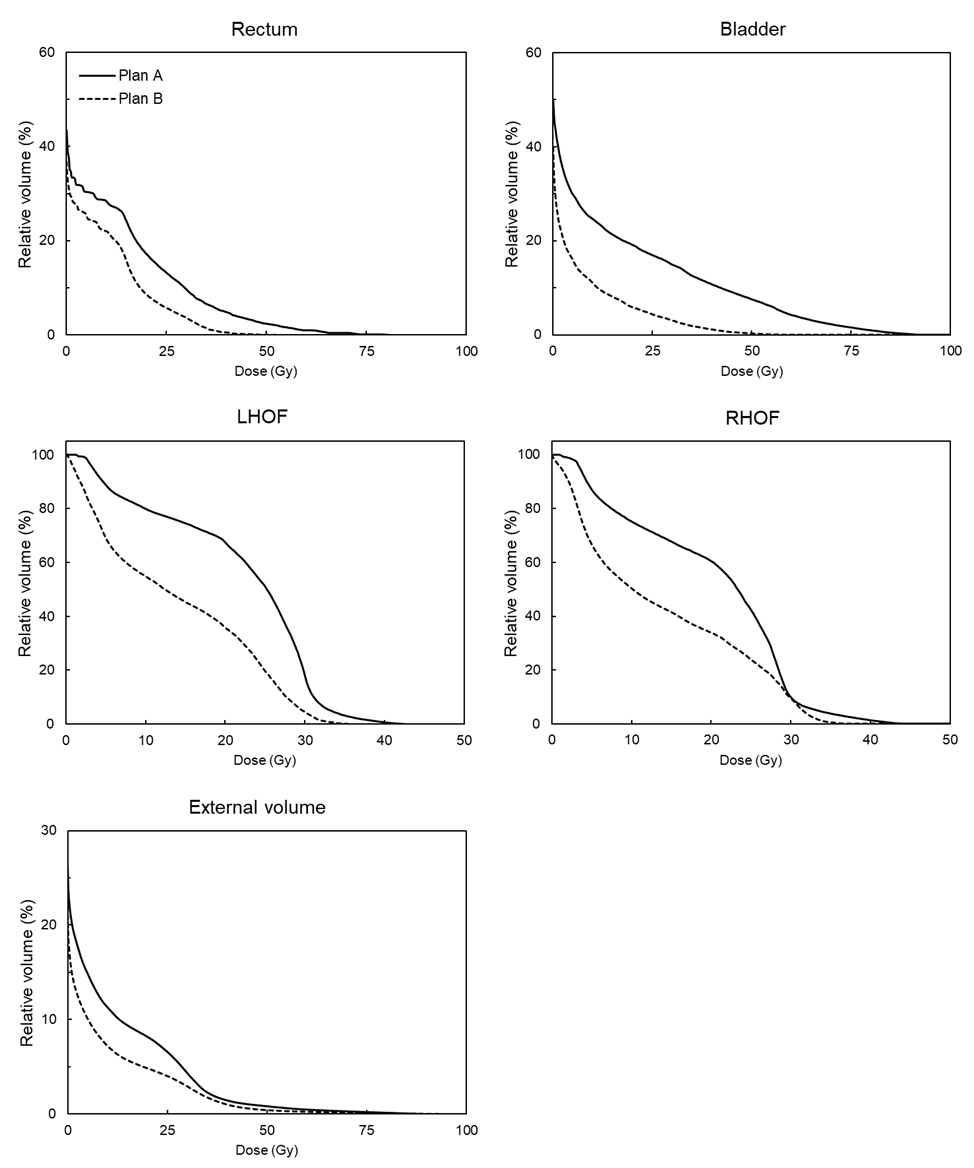


Figure 15 OAR DVH for Patient 3, isoeffective Plans A and B with a <TCP>=0.95.


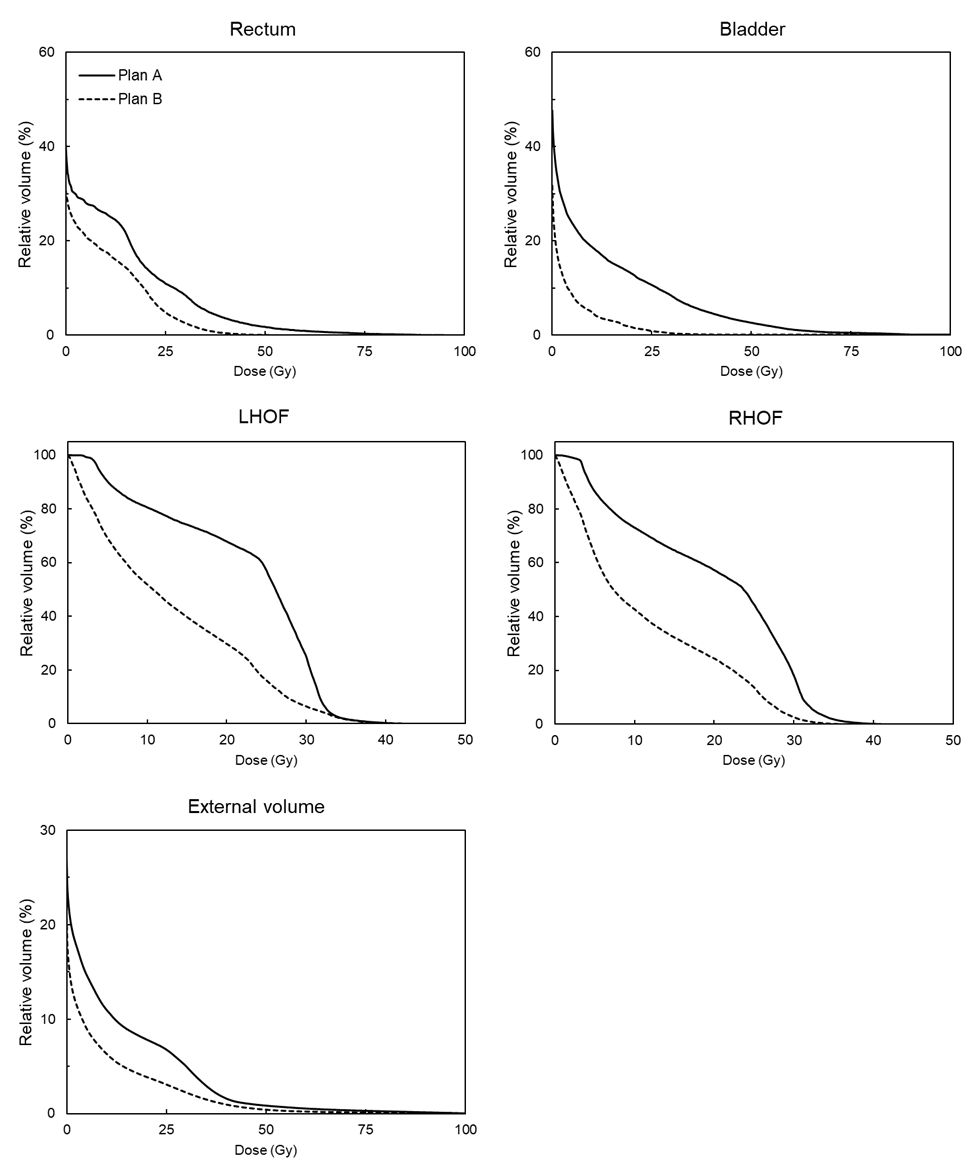


Figure 16 OAR DVH for Patient 4, isoeffective Plans A and B with a <TCP>=0.95.


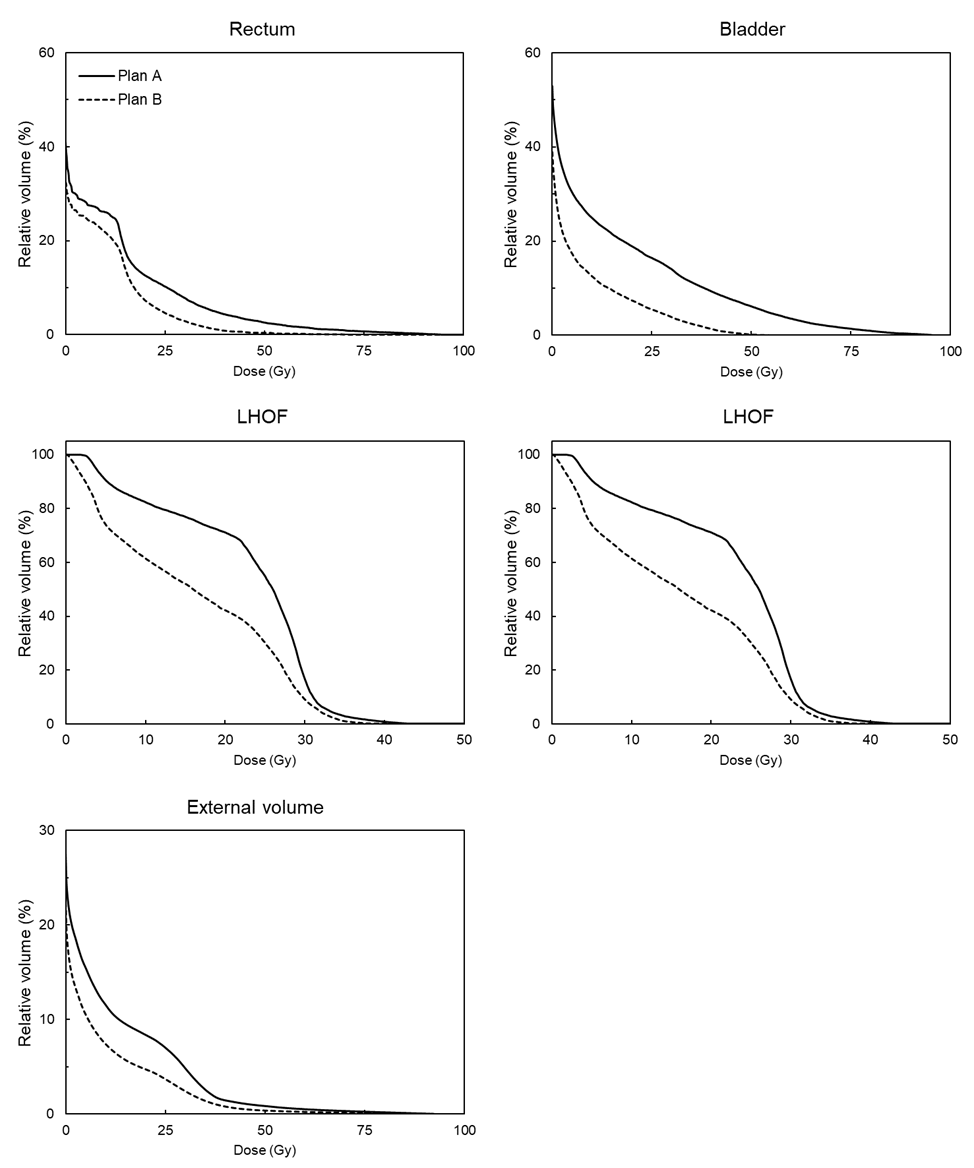


Figure 17 OAR DVH for Patient 5, isoeffective Plans A and B with a <TCP>=0.95.

**Rectal & bladder <NTCP>**

| <NTCP> was calculated as: $\left\langle NTCP \right\rangle=\frac{1}{1+\left( \frac{TD50}{\left\langle EUD \right\rangle} \right)^{x}}$ |
| --- |

where $x=\frac{4}{m\sqrt{2\pi}}$ and *TD50* is the uniform dose which will lead to complication in 50% of the population.

For rectal toxicity (grade ≥1 rectal bleeding): TD50=78.4 Gy, m=0.108 (Söhn et al. 2007)

For bladder toxicity (grade ≥1 GU toxicity): TD50= 80 Gy, m=0.11 (Emami et al. 1991; Luxton et al. 2004).


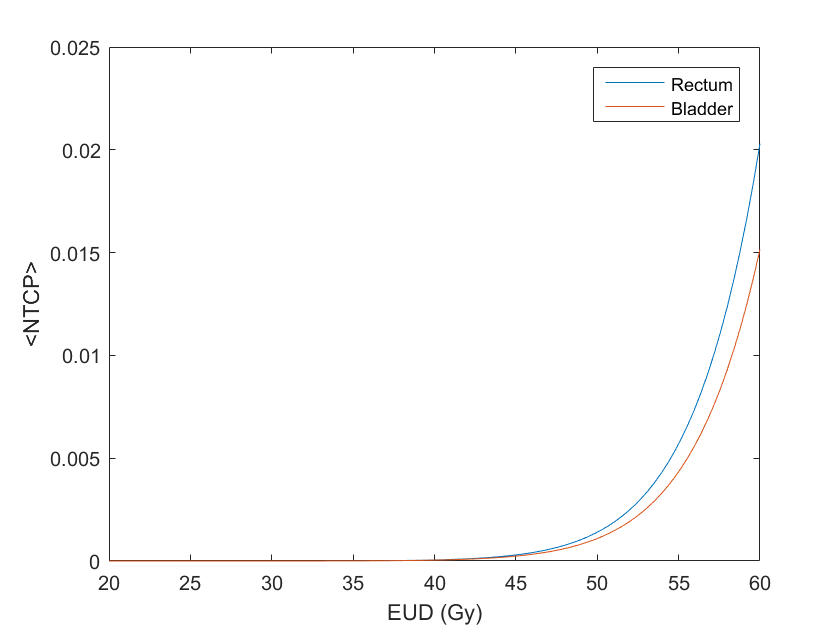


Figure 18 Rectal and bladder NTCP demonstrating a large variance for the <EUD> range observed in Plan A (Table 5 of the manuscript).

| **<NTCP>** | **Patient 1** | | **Patient 2** | | **Patient 3** | | **Patient 4** | | **Patient 5** | | **Mean change**  **(mean % change)** | ***p*** |
| --- | --- | --- | --- | --- | --- | --- | --- | --- | --- | --- | --- | --- |
|  | **Plan A** | **Plan B** | **Plan A** | **Plan B** | **Plan A** | **Plan B** | **Plan A** | **Plan B** | **Plan A** | **Plan B** |  |  |
| **Rectum** | 6.4E-04 | 4.4E-07 | 3.3E-04 | 1.5E-07 | 7.1E-06 | 8.0E-09 | 1.2E-05 | 5.0E-09 | 4.6E-05 | 6.5E-08 | -2.1E-04  (-99.9%) | **0.17** |
| **Bladder** | 4.3E-04 | 6.8E-09 | 1.4E-04 | 1.3E-08 | 1.7E-04 | 1.5E-08 | 9.9E-06 | 1.2E-11 | 1.5E-04 | 1.6E-08 | -1.8E-04  (-99.9%) | **0.06** |

Table 1 NTCP comparison for isoeffective Plans A and B
